# Effects of an Automated External Defibrillator With Additional Video Instructions on the Quality of Cardiopulmonary Resuscitation

**DOI:** 10.3389/fmed.2021.640721

**Published:** 2021-03-17

**Authors:** Florian Ettl, Eva Fischer, Heidrun Losert, Dominik Stumpf, Robin Ristl, Kurt Ruetzler, Robert Greif, Henrik Fischer

**Affiliations:** ^1^Department of Emergency Medicine, Medical University of Vienna, Vienna, Austria; ^2^Department of Anaesthesiology and Intensive Care, Klinik Donaustadt, Vienna, Austria; ^3^Department of Anaesthesia and Intensive Care, Ordensklinikum Linz - Hospital of the Sisters of Charity, Linz, Austria; ^4^Center for Medical Statistics, Informatics and Intelligent Systems, Medical University of Vienna, Vienna, Austria; ^5^Department of Outcomes Research, Cleveland Clinic, Cleveland, OH, United States; ^6^Department of Anaesthesiology and Pain Medicine, Inselspital, Bern University Hospital, University of Bern, Bern, Switzerland; ^7^Medical School, Sigmund Freud Private University, Vienna, Austria

**Keywords:** resuscitation, basic life support, automated external defbrillator, quality, video instructions

## Abstract

**Aim of the Study:** The aim was to compare cardiopulmonary resuscitation (CPR) quality of an automated external defibrillator (AED) with and without additional video instruction during basic life support (BLS) by laypersons.

**Methods:** First-year medical students were randomized either to an AED with audio only or audio with additional video instructions during CPR. Each student performed 4 min of single-rescuer chest compression only BLS on a manikin (Ambu Man C, Ballerup, Denmark) using the AED. The primary outcome was the effective compression ratio during this scenario. This combined parameter was used to evaluate the quality of chest compressions by multiplying compressions with correct depth, correct hand position, and complete decompression by flow time. Secondary outcomes were percentages of incomplete decompression and hand position, mean compression rate, time-related parameters, and subjective assessments.

**Results:** Effective compression ratio did not differ between study groups in the overall sample (*p* = 0.337) or in students with (*p* = 0.953) or without AED experience (*p* = 0.278). Additional video instruction led to a higher percentage of incorrect decompressions (*p* = 0.014). No significant differences could be detected in time-related resuscitation parameters. An additional video was subjectively rated as more supporting (*p* = 0.001).

**Conclusions:** Audio–video instructions did not significantly improve resuscitation quality in these laypersons despite that it was felt more supportive. An additional video to the verbal AED prompts might lead to cognitive overload. Therefore, future studies might target the influence of the video content and the potential benefits of video instructions in specific populations.

## Introduction

Out-of-hospital cardiac arrest is among the leading causes of death in Europe (1). In the EMS response to a cardiac arrest, ventricular fibrillation is the initial cardiac rhythm in up to 25–50% of patients (1). Early recognition of cardiac arrest, early initiation of high-quality chest compression, and early defibrillation using an automated external defibrillator (AED) remain the cornerstone to increase patient survival (1–4). The fear to perform cardiopulmonary resuscitation (CPR) and the incorrect use of AED are bystanders' barriers to provide these life-saving interventions (5–8). Commercially available AEDs offer verbal instructions on its correct use and how to perform CPR. AEDs support untrained people in providing the most probable effective CPR (9). However, previous studies reported that the quality of CPR and the time until the first shock was given by the AED varied significantly among different types of AEDs (9–12). Several studies investigated the influence of audio prompts on the effectiveness of laypersons' CPR compared with CPR without any audio instructions. Adaptions and evaluation of given AED instruction protocols were suggested to improve the CPR quality of the AED user (13, 14). Video instructions are used in various fields primarily for educational purposes (15–20). The addition of visual instructions to conventional AED audio commands offers a possibility to further develop AED instructions and, therefore, may improve CPR quality.

The aim of our study was to investigate the differences in CPR quality and subjective rating between AED audio–video prompts and audio prompts only. We hypothesized that adding additional video instructions to the conventional AED audio instructions improves CPR quality measured by effective compression ratio (ECR).

## Materials and Methods

The study was performed at the Medical University of Vienna, Austria, between October and December 2016. The requirement for ethical approval was waived by the Ethics Committee of the Medical University of Vienna (No. 1945/2016).

In this open randomized, controlled parallel group study, we enrolled medical students at the beginning of the obligatory first aid course during the first semester of medical school. In order to include laypersons only, professionally trained individuals like emergency medicine technicians, paramedics, or nurses with intermediate or advanced life support training, according to the European Resuscitation Council, were excluded from the study. Further exclusion criteria were age < 18, inadequate German language skills, and injuries that made it impossible to adequately perform CPR. The students were informed that study participation was voluntary and would not influence their grades of their first aid course.

After providing written informed consent, medical students were stratified into two groups: (I) students with AED experience (who had a first aid course with AED training before starting to study medicine) and (II) students without any AED experience (never took a first aid course or participated in a first aid course without AED training).

After stratification, students were randomly assigned equally to one of two groups: (1) AED group with audio–video prompts and (2) AED group with audio prompts only, using the Randomizer for Clinical Trials (Version 1.8.1, Department of Statistics, Medical University of Vienna, Vienna, Austria; https://www.meduniwien.ac.at/randomizer/web/login.php).

After randomization, when starting the study, students were told to switch on the AED and this was the time the study started. The students followed the instructions of the device. The assessment and chronological documentation of events (turning on the AED, administration of shocks, start and stop of chest compressions) started at the same time. Then, the defibrillation pads were applied accordingly on the manikin, and defibrillation had to be administered as programmed for this study before the students started with single-rescuer chest compression only CPR according to the ERC 2015 guidelines (1). After 2 min of chest compressions, the AED indicated a second rhythm analysis and a further shock was advised to be administered. The study scenario was terminated after two shocks and 4 min of chest compressions in total, and study participants filled in a questionnaire. The case report form containing the sequence and the documented time points can be found as a supplement. Due to the high number of participants, the testing was performed with the same setup simultaneously in two rooms. Only one study participant and one investigator were present in the room during the study. Obviously, participants and the evaluating investigators could not be blinded. The data-handling staff performing data acquisition, data entry, and data analysis were blinded to the group assignment of the study participants.

We used the Ambu Man C (Ambu, Ballerup, Denmark) with medium thorax resistance to assess CPR quality. The accuracy of the manikins and software measurements was assessed prior to the study as described earlier (21). Differences in sensitivity between the two identical manikins used were negligible. The manikins were placed in supine position on an antislip layer on an even floor to ensure accurate measurement.

Time-related parameters (e.g., from scenario start until placement of AED pads, or until administration of the first shock) were measured *via* a stopwatch by trained investigators. Data were collected on a computer (Fujitsu Siemens, Amilo PA 1510) using the Ambu CPR software (version 2.3.9, Ambu, Ballerup, Denmark). The AEDs (Lifeline View AED Trainer DDU-2000, Defibtech, Guilford, USA) used in the study were adjusted during the study to indicate shockable rhythms only. This AED offers a display providing video instructions in addition to the audio prompts. The AED was placed next to the manikin's head to offer a good view on the display. To create the reproducible conditions, two identical models were used in the two interventional groups. The display in the voice-only group was covered with a non-transparent adhesive lamination.

The primary outcome parameter was the ECR. This ratio describes the general performance of CPR considering correct hand positioning, chest compression depth, and complete decompression multiplied by the flow time fraction as described previously (22). Secondary outcome parameters were effective compressions (defined by compressions with correct hand positioning, chest compression depth, and complete decompression), mean compression rate and depth, percentages of incomplete decompression, and incorrect hand position. Time-related outcomes were time from activation of the device until the placement of the pads, time until first shock delivery, time until first chest compression, and peri-shock pause (time from last chest compression before the second shock till the first compression after). Absolute and relative flow times were also recorded.

All study participants were asked to evaluate their subjective experience using a questionnaire concerning AED use and their personal assessment of CPR quality after testing on a 10-point Likert scale (10 points representing the highest and 0 representing the lowest rating). The last two questions evaluated the usefulness of the video instruction during resuscitation and were answered by the participants of the audio–video group only. The full questionnaire can be found as Electronic Supplementary Material.

### Statistics

The main outcome ECR was compared between the two randomization groups (audio–video prompt AED vs. audio-only prompt AED) using a Wilcoxon rank sum test at a pre-specified significance level of 0.05. Based on a previous investigation, the sample size was calculated under the assumption that the standard deviation of ECR would be 0.3 (23). A sample size of 202 per group was found to result in 90% power for the Wilcoxon test, assuming a mean difference of 0.1 between the groups and normally distributed data.

Further, ECR and a set of secondary outcomes were compared between the two randomization groups as well as between subjects with and without previous AED experience. Metric variables were described by median and interquartile range (presented in square brackets) and were compared between groups using Wilcoxon tests. For the single binary variable indicating appropriate mean compression rate, absolute and relative frequencies were calculated, and the groups were compared using a chi-squared test. Tests for interactions between the randomization group and AED experience were calculated from linear or logistic regression models, as appropriate, including the randomization group, the binary indicator of previous AED experience, and the interaction of both factors as predictor variables. The hypothesis tests for secondary outcomes are considered exploratory and no multiple testing correction was applied.

## Results

**Figure 1:**
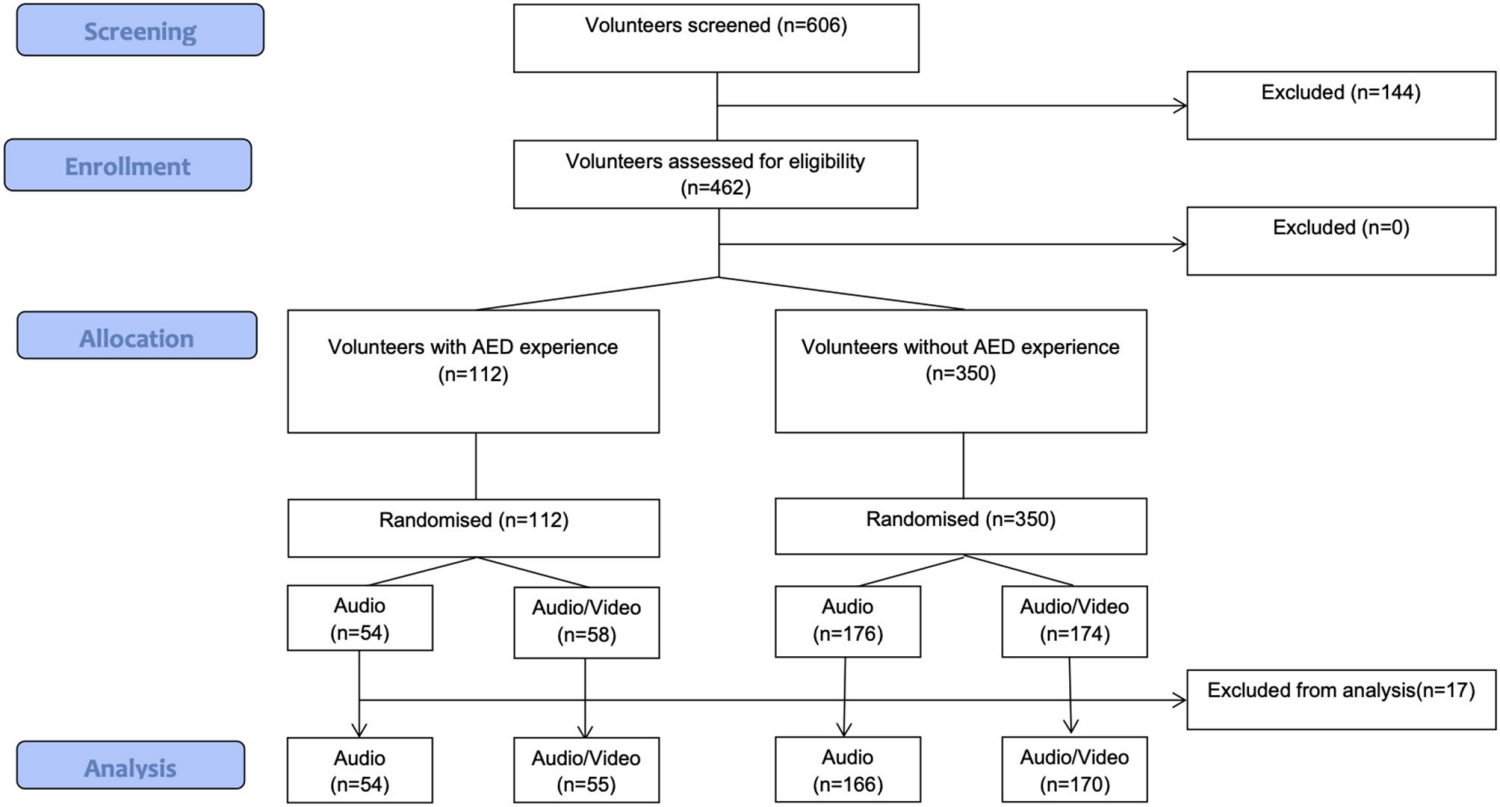
Participant flowchart.

A total of 606 medical students were screened for this study. Out of those, 462 were included for randomization and 144 were not eligible due to exclusion criteria. We accepted all available students even if the pre-calculated sample size was lower. One hundred and twelve study participants used an AED before, whereas 350 participants had no AED experience at all. After the exclusion of 17 cases due to missing data, 54 trained participants in the audio group and 55 trained participants in the audio–video group were analyzed. In the group without previous AED experience, 166 participants received audio instruction and 170 received audio–video instruction (Figure 1).

The gender distribution between groups was equal and no significant difference in age was found. Participants' characteristics are reported in Table 1.

**Table 1:** Demographics summarized as median and IQR or absolute and relative (%) frequencies.

	**Audio (*n* = 220)**	**Audio–video (*n* = 225)**
Female, *n* (%)	151 (69)	146 (65)
Weight (kg)	61 (55–70)	63 (56–73)
Height (cm)	172 (167–179)	172 (167–179)
BMI	20.9 (19.5–22.9)	21.2 (19.8–23.3)
Age (years)	20 (19–21)	20 (19–21)
**Last basic life support (BLS) course within** ***n*** **(%)**		
<6 months ago	8 (4)	14 (6)
6–12 months ago	11 (5)	6 (3)
12–24 months ago	12 (5)	15 (7)
>24 months ago	23 (10)	20 (9)
No BLS course	166 (75)	170 (76)

The effective compression ratio in the audio group did not differ from that in the audio–video group in either AED experience stratum. No difference was found between the ECR of participants with and without AED experience (Table 2). Audio–video instruction led to a higher percentage of incorrect decompressions (16.2 vs. 40.4%, *p* = 0.014).

**Table 2:** Outcomes are summarized as median and IQR or absolute and relative (%) frequencies.

	**Audio (*n* = 220)**	**Audio–video (*n* = 225)**	p^*^	**Untrained (*n* = 336)**	**Trained (*n* = 109)**	p^*^	*p* int^**^
**Chest compression parameters**							
Effective compression ratio (ECR)	0.01 (0–0.15)	0.01 (0–0.13)	0.337	0.01 (0–0.12)	0.03 (0–0.24)	0.063	0.659
Effective compressions (%)	1.5 (0–17.6)	1.1 (0–15.1)	0.296	1.1 (0–14.0)	3.5 (0–27.2)	0.069	0.683
Compression rate (min^−1^)	100 (99–102)	100 (100–101)	0.250	100 (99–102)	100 (100–101)	0.651	0.405
Compression depth (mm)	52 (42–60)	51 (41–59)	0.352	51 (41–59)	53 (44–60)	0.050	0.633
Incorrect decompressions (%)	16.2 (1.0–68.5)	40.4 (2.3–87.0)	0.014	27.4 (1.2–79.8)	29.3 (1.7–76.7)	0.884	0.622
Incorrect pressure point (%)	2.4 (0–39.8)	1.8 (0–38.5)	0.993	4.3 (0–41.1)	0.3 (0–23.0)	0.017	0.282
Correct mean compression rate	125 (57%)	133 (59%)	0.694	198 (60%)	60 (55%)	0.547	0.396
Correct mean compression depth	69 (31%)	76 (34%)	0.658	104 (31%)	41 (38%)	0.241	0.855
**Time-related parameters**							
Flow time (s)	203 (202–205)	204 (202–205)	0.090	203 (202–205)	204 (203–206)	0.000	0.487
Flow time fraction (%)	84.7 (84.1–85.5)	85.0 (84.3–85.5)	0.090	84.7 (84.1–85.3)	85.1 (84.5–85.3)	0.000	0.487
Time till first shock (s)	69 (65–72)	67 (64–76)	0.258	69 (65–74)	67 (64–71)	0.031	0.494
Peri-shock pause (s)	36 (35–37)	36 (35–37)	0.465	36 (35–37)	36 (35–37)	0.000	0.418

*ECR: effective compression ratio was defined as effective compressions (%) multiplied by flow time (%)*.

*Effective compressions were defined as compressions with correct depth (50–60 mm), correct hand position, and complete decompression*.

*Participants with correct mean compression rate were defined as a mean compression rate between 100 and 120 min^−1^*.

*Subjects with correct mean compression depth were defined as mean compression depth between 50 and 60 mm*.

*Flow time was defined as the sum of all periods during which chest compressions were performed*.

*Absolute hands-off time was defined as the sum of all periods without chest compressions*.

*Time till first shock (s): time from scenario start until administration of the first shock*.

*Peri-shock pause (s) = time from last chest compression before the second shock till the first compression after*.

* *p-values for the comparisons of two groups were calculated using the Wilcoxon test*.

** *Interactions between the effect of audio–video vs. audio and AED experience were tested from linear or logistic regression models containing both grouping variables and their interaction as predictors*.

Participants with previous AED experience performed significant deeper compressions than participants without AED experience [51 (41–59) vs. 53 (44–60) mm, *p* = 0.050]. The percentage of compressions with incorrect pressure point was comparable for all the groups (Table 2). Comparing all four groups showed no significantly different results for any compression-related parameter.

Participants with AED experience showed minimal longer flow time compared with participants without AED experience [204 (203–206) vs. 203 (202–205) s, *p* < 0.001]. There was no significant difference in time needed to place the defibrillator pads and to the first chest compression after shock administration between the audio and the audio–video groups. Also, the peri-shock pause did not vary significantly between these groups (Table 2).

Participants with AED experience administered the first shock earlier [67 (64–71) vs. 69 (65–74) s, *p* = 0.031].

The subjective rating of resuscitation quality did not correlate with the objective performance measured by ECR (rho = 0.007, *p* = 0.877). The study group with audio–video instructions rated subjectively the instructions on the Likert scale as more understandable [9 (8–10) vs. 10 (9–10), *p* < 0.001] and supporting [9 (8–10) vs. 10 (9–10), *p* < 0.001]; 89% of participants with and 98% of participants without previous AED experience felt supported by the audio–video instructions (*p* = 0.009). An interesting finding resulting from the analysis of the questionnaire is that inexperienced participants rated themselves on the 10-point Likert scale and stated that they paid significantly more attention to the video than trained individuals [8 (7–9) vs. 7 (5–8), *p* < 0.001].

## Discussion

The results of this manikin study in first-year medical students who were considered as laypersons in CPR indicate that the addition of video instructions to audio AED instructions during chest compression only CPR does not improve the overall quality of CPR.

Incomplete decompressions appeared more often in the group with visual instructions. Interestingly, neither feedback devices nor the audio–video instructions like in this study could improve the percentage of incomplete decompressions (21).

CPR with the assistance of AED instructions can reassure and guide even untrained laypersons through chest compressions and shock application (14). By means of the subjective assessment, we wanted to gain a deeper insight into the participants' thoughts concerning the effect of the video during CPR instructions. Interestingly, these audio–video instructions were experienced as more supportive in understanding what to do, and untrained participants paid more attention to the video instructions. This is supported by earlier studies: videos influence behavior, compliance, and acceptance of interventions (20). However, oftentimes, the subjective impression of participants in the audio–video group did not result in objectively measured CPR performance.

Chest compression parameters did not differ between participants with and without AED experience. Others showed that AED experience leads to better time-related variables (flow time, time till first shock, and peri-shock pause) (24). Due to the fact that first aid courses greatly differ in their quality, especially as far as the application of an AED is concerned, we could not sufficiently explore the participants' experience concerning its use. Therefore, we are unable to shed light on this difference.

Differences in AED design and audio prompts are influencing CPR quality (9, 11). In our study, we could not reproduce an improved CPR performance with the addition of video prompts, even if such an additional video instruction seems intuitive as well as helpful. However, as the human working memory has its limitation following the cognitive load theory, the addition of more prompts to the audio might be overwhelming for laypersons in such a stressful situation as CPR, even if that happens during simulation but in the setting of a study (25). As so often cited also in this situation, less might be more. On the other hand, having in mind what was mentioned before, future studies or developments of AED might focus more on specific content and details of the video instructions. Another development might be the combination of video prompts with audio–video feedback device to improve CPR quality during action. Finally, we do not know if certain groups might benefit more than others from visual instructions, e.g., age groups, training levels, or persons with a disability (e.g., deaf-mute).

Certainly, a limitation of this study is that in our setting we were not able to assess the influence, strength, and weaknesses of the content of this specific instructional video as it was given and predetermined by the providing device distributor.

We included a sufficient and high number of study participants and first-year medical students who were at the start of their medical education. We considered them as laypersons, as they did not receive any specific emergency medical education prior to this study. Therefore, the results of this study seem generalizable to laypersons with a minimum of CPR education.

The addition of video prompts to conventional audio prompts did not lead to improved CPR quality. Further studies might investigate specific video content possibly in relation to CPR performance feedback or video prompts for specific target groups of rescuers. Another issue to study might be the influence of video prompts on overcoming the fear to perform CPR and how it might improve the willingness to perform CPR using an AED.

## Data Availability Statement

The raw data supporting the conclusions of this article will be made available by the authors, without undue reservation.

## Ethics Statement

The study was performed at the Medical University of Vienna, Austria between October and December 2016. The requirement for ethical approval was waived by the Ethics Committee of the Medical University of Vienna (No: 1945/2016). Participants provided written informed consent.

## Author Contributions

FE: conceptualization, methodology, investigation, project administration, writing—original draft, and writing—review and editing. EF: methodology, investigation, and writing—review and editing. HL: resources, investigation, and writing—review and editing. DS: software, data curation, formal analysis, and writing—review and editing. RR: methodology, formal analysis, and writing—review and editing. KR: formal analysis and writing—review and editing. RG: conceptualization, methodology, supervision, and writing—review and editing. HF: conceptualization, methodology, project administration, resources, supervision, and writing—review and editing. All authors contributed to the article and approved the submitted version.

## Conflict of Interest

RG is the ERC Director of Training and Education, ILCOR Task Force Chair Education, Implementation, and Teams; Associate Editor of Resuscitation Plus, European Journal of Anesthesiology; and Editor-in-Chief of Trends in Anaesthesia and Critical Care. The remaining authors declare that the research was conducted in the absence of any commercial or financial relationships that could be construed as a potential conflict of interest.
