# Supplementary material for: Effects of an Automated External Defibrillator With Additional Video Instructions on the Quality of Cardiopulmonary Resuscitation
Source: Front Med (Lausanne). 2021 Mar 17;8:640721. doi: 10.3389/fmed.2021.640721 (PMC8009965; doi:10.3389/fmed.2021.640721)
Supplement: Supplementary file 1 [file Image_1.pdf]

ID: ☐ test station: ☐

AED: Audio-Video vs. Audio

Sex:	O m O w
Weight (kg):	
Height (cm):	
BMI (to be filled in by the study staff):	
Age:	
Have you ever performed CPR in real life?	O Yes, without AED<br>O Yes, **with** AED<br>O No
Did you already attend a CPR training/first aid course?	O Yes, without using an AED<br>O Yes, an **AED** was **used**<br>O No
When was the last time you attended a CPR training/first aid course in which an **AED** was **used**?	O never<br>O within the last 6 months<br>O 6 to 12 months ago<br>O 12 to 24 ago<br>O more than 24 months ago

Randomisation number **proband** (to be filled in by the study staff):

Audio-Video ☐

Audio ☐

ID: test station:

Chronological sequence of CPR

min:sec	Event
00:00	Turning on the AED
	Pad placement — correct ☐ not correct ☐
	1. Shock
	Start chest compressions
	Stop chest compressions
	2. Shock
	Start chest compressions
	Finish

ID: ☐ test station: ☐

Subjective evaluation

Question 1
How simple/challenging was performing CPR using an Automated External Defibrillator from your point of view?

1	2	3	4	5	6	7	8	9	10
Highly demanding									fairly simple

Question 2
How intelligible were the Automated External Defibrillator's instructions during the course of performing CPR?

1	2	3	4	5	6	7	8	9	10
incomprehensible									highly intelligible

Question 3
To which degree were the Automated External Defibrillator's instructions supporting you during the course of performing CPR?

1	2	3	4	5	6	7	8	9	10
Too little									highly supportive

Question 4
How would you rate the quality of your chest compressions with respect to effectiveness?

1	2	3	4	5	6	7	8	9	10
Rather bad									very good

Question 5
How challenging was the resuscitation process for you?

1	2	3	4	5	6	7	8	9	10
Not at all									To a maximum

If there were **video-instructions** available, please answer the following questions as well:

Question 6
How much attention did you pay to the video instructions?

1	2	3	4	5	6	7	8	9	10
No attention at all									a lot of attention

Question 7
Was the video supportive concerning your emergency efforts?

O Yes O No

Thank you for participating – your study staff
